# Exosome Circuitry During (De)(Re)Myelination of the Central Nervous System

**DOI:** 10.3389/fcell.2020.00483

**Published:** 2020-06-16

**Authors:** Helena S. Domingues, Ana Mendanha Falcão, Inês Mendes-Pinto, António J. Salgado, Fábio G. Teixeira

**Affiliations:** ^1^Life and Health Sciences Research Institute (ICVS), School of Medicine, University of Minho, Braga, Portugal; ^2^ICVS/3B’s Associate Laboratory, PT Government Associate Laboratory, Braga/Guimarães, Portugal; ^3^International Iberian Nanotechnology Laboratory (INL), Braga, Portugal

**Keywords:** Exosomes, myelin, axon-oligodendrocyte unit, demyelinating diseases, biomarkers, therapeutic vehicles, mesenchymal stem cells, secretome

## Abstract

Reciprocal neuron–glia cell communication is fundamental for the proper function of the nervous system. Oligodendrocytes are the myelinating cells of the central nervous system (CNS) that insulate and provide trophic support to neurons. This effective interaction is crucial not only for myelination but also for long-term axonal survival and neural connectivity. In recent years, exosomes have been portrayed as key players in intercellular interaction in the context of the healthy and diseased CNS. They act as communicating vehicles, true attachés operating between neurons and glial cells. Despite the complex exosome circuitry within the nervous system, experimental evidence supports the role of exosomes in modulating myelination. Oligodendrocytes secrete exosomes in response to neuronal signals in an electric activity-dependent manner. These released exosomes are then internalized by neurons, contributing to their integrity and activity. In turn, neurons secrete exosomes to control the communication between them and with myelinating cells in order to regulate synaptic function in neuronal development, myelin maintenance, and neuroregeneration. In this review, we provide a critical view of the current understanding on how exosomes, either from CNS-resident cells or from the periphery, contribute to the formation and maintenance of myelin and, additionally, on how the differential content of exosomes in normal and pathological conditions foresees the use of these nanovesicles as putative diagnostic and/or therapeutical agents in white matter degeneration-associated diseases.

## Introduction

The CNS myelination mechanisms emerge from intercellular communication events that occur during development and underlie dramatic morphological alterations. Oligodendrocytes produce myelin, a lipid-rich membrane that wraps axons providing insulation and metabolic support. In fact, the myelin sheet is metabolically coupled with its subjacent axon through cytoplasmic-rich pathways and myelinic channels that allow movement of macromolecules into the periaxonal space. One such example is the oligodendrocyte-derived lactate that is metabolized within myelinated tracts for mitochondrial ATP production (Funfschilling et al., 2012; Simons and Nave, 2016). A considerable body of research has focused on the identification of axonal- and oligodendrocyte-derived signals necessary for CNS myelination (extensively reviewed by Chong and Chan, 2010). In the absence of neuronal biochemical instructions but with appropriate biophysical cues, oligodendrocytes have an intrinsic property to produce specialized myelin sheath membranes and generate an appropriate 3D myelin architecture (Lee et al., 2012, 2013; Bechler et al., 2015; Domingues et al., 2018). However, the myelination process is permissive to neural activity dependent on action potentials capable of stimulating neighboring oligodendrocytes to myelinate axons (Gibson et al., 2014; McKenzie et al., 2014; Etxeberria et al., 2016). This interaction between axon and oligodendrocyte in the internodal, paranodal, and juxtaparanodal regions is highly dynamic and mediated by cell–cell adhesion and tight junction molecular complexes (Devaux and Gow, 2008; Elazar et al., 2019). Yet, cell contact-independent mechanisms also take place and are mediated by paracrine signaling. In this context, molecular cues can be released in their soluble form or confined into extracellular vesicles (EVs) to elicit various responses at the targeted cell (Colombo et al., 2014). EVs are a heterogeneous group of membrane vesicles of endosomal or plasma membrane origin, from which exosomes constitute the smallest type of EVs, ranging in size between ∼30 and 150 nm. Exosomes were first described in the 1980s as small EVs seen by electron microscopy and secreted by reticulocytes (Harding et al., 1983; Pan and Johnstone, 1983; Pan et al., 1985; Johnstone et al., 1987). Of endosomal origin, exosomes are generated by the trafficking of multivesicular bodies (MVBs) that are released to the extracellular environment upon fusion with the plasma membrane (extensively reviewed by Colombo et al., 2014). All cells of the CNS release exosomes (Holm et al., 2018; Krämer-Albers and Hill, 2018), including neurons (Fauré et al., 2006), oligodendrocytes (Krämer-Albers et al., 2007; Fitzner et al., 2011), microglia (Potolicchio et al., 2005), and astrocytes (Taylor et al., 2007). These nanoscale vesicles are secreted both in homeostatic and pathogenic conditions and work as messengers in intercellular communication. Exosome cargo, consisting of membrane and cytosolic proteins, lipids, and nucleic acids, are released and targeted into a recipient cell in a specific manner to induce changes in its physiology, as discussed in higher detail in the next sections. In the context of the CNS, experimental evidence suggests that exosomes are crucial players not only in neuron–neuron (Chivet et al., 2014) but also in neuron–glia (Men et al., 2019) communication, with large repercussions in myelin development and repair (Krämer-Albers and Hill, 2018). If that is the case, what is their effective contribution to myelin biogenesis and maintenance? Do these messengers work in an iterative process? That is, do they work in feedback loops where the production of both oligodendrocyte- and neuron-derived exosomes is orchestrated in a synchronous manner to promote a proper neural circuitry? Can myelination be modulated by exosomes produced by other CNS-resident cells or non-resident cells? Moreover, can exosomes derived from young or stem cell microenvironments promote myelination and, consequently, neuroregenerative effects? If so, can we engineer artificial exosomes with specific contents to serve as a CNS neuroregenerative biopharmaceutical asset? Here, we review what is the current state of the art on how myelination is influenced by exosomes, not only through the complex interaction between CNS-resident cells but also by exosomes of cells from peripheral origin. We also discuss the existing gaps and how the knowledge of this expanding field can be applied in human health through the use of exosomes as potential diagnostic biomarkers and therapeutical agents.

## Exosomes in Myelin Development and Maintenance

Myelin is a highly specialized membrane with a unique structure and composition. In comparison to other membranes, myelin possesses in its dry weight an opposite proportion of lipids to proteins with more than 70% of lipids and most of its protein composition is myelin-specific. Myelin basic protein (MBP) and proteolipid proteins (PLP) are the most abundant myelin proteins found in the compact myelin fraction (Simons and Nave, 2016). Trajkovic et al. (2006) found that oligodendrocytes produce and sort myelin membrane upon neuronal request through regulation of myelin trafficking. Specifically, PLP is internalized and stored in late endosomes/lysosomes (LEs/Ls) of multivesicular and multilamellar appearance and the transport of PLP from LEs/Ls to the plasma membrane is triggered by neuronal signals (Trajkovic et al., 2006). In 2007, it was shown for the first time that oligodendrocytes secrete exosomes into the extracellular space in a calcium dependent manner (Krämer-Albers et al., 2007). Another study identified the sphingolipid ceramide to be required for intraendosomal membrane transport and exosome formation in oligodendrocytes (Trajkovic et al., 2008). In these studies, oligodendroglial exosomes contained major amounts of myelin proteins, such as PLP (Krämer-Albers et al., 2007; Bakhti et al., 2011), MBP, myelin oligodendrocyte glycoprotein (MOG), and 2’3’-cylic-nucleotide-phosphodiesterase (CNPase), along with myelin-specific lipids and other proteins such as 14-3-3 proteins, heat shock proteins, dihydropyrimidinase-related proteins, and peroxiredoxin. These chaperones and enzymes involved in the management of oxidative stress led the authors to speculate that these molecules may be involved in providing trophic support to axons (Krämer-Albers et al., 2007; Trajkovic et al., 2008; Bakhti et al., 2011). Additionally, Bakhti et al. (2011) observed that exosomes derived from oligodendrocytes have an autoinhibitory effect in cell differentiation by reducing oligodendrocyte surface expansion, in a process dependent of actomyosin contractility. When in co-cultures with neurons, one or more factors in the neuronal conditioned medium inhibited the release of exosome-like vesicles from oligodendrocytes, suggesting that neurons control myelin membrane biogenesis by regulating the release of oligodendroglial autoinhibitory exosomes. In another study with cell contact-independent co-cultures of neurons and oligodendrocytes, Frühbeis et al. (2013) showed that neuronal glutamate was able to trigger the release of oligodendroglial exosomes in a process mediated by calcium entry through glial NMDA and AMPA receptors. Using microfluidic chambers, the authors verified that oligodendroglial exosomes were internalized by neurons by endocytosis at axonal and somatodendritic sites and improved cell viability under stress conditions (Frühbeis et al., 2013). Later, the same authors identified an extensive spectrum of positive effects of oligodendroglial exosomes on neuronal physiology, namely neuronal survival and antioxidant activity upon oxygen-glucose deprivation, increased neuronal firing rate, and transcriptional regulation (Fröhlich et al., 2014). However, the specific molecules mediating such effects were not identified. In a zebrafish myelination model, the inhibition of neuronal synaptic vesicle release affected the myelinating capacity of oligodendrocytes during their initial period of sheath formation (Mensch et al., 2015). This work supports the concept of glutamate-dependent myelination observed *in vitro*. It remains to be seen whether in this model exosomes are active participants in promoting neuron-oligodendrocyte communication-mediated myelination.

Similar mechanisms of exosome release seem to operate in myelinating cells of the peripheral nervous system (PNS) but the exosome content of Schwann cells (SC) differs from the ones described in oligodendrocytes. There is evidence that after axotomy SC dedifferentiate and secrete exosomes that are specifically internalized by dorsal root ganglia (DRG) axons and promote axonal regeneration after axotomy (Lopez-Verrilli et al., 2013). These exosomes contained common exosome markers such as CD63, Tsg101, Hsp70, Hsp90, and flotillin-1. In addition, they contained p75-neurotrophin receptor (p75NTR), characteristic of a dedifferentiated SC phenotype and usually expressed after nerve damage. Recently, it was shown that upon DRG-derived glutamate stimulus, Schwann cells released exosomes in a calcium-dependent manner, which increased nerve cells activity (Hu et al., 2019). Characterization of exosomes from Schwann cells-conditioned medium by proteomics revealed the presence of molecules that are closely related to axon regeneration, such as carboxypeptidase E (CPE), fatty acid-binding protein (FABP5), fibronectin, flotillin-2, major vault protein (MVP), monocarboxylate transporter 1 (MCT1), neuropilin-2 (NRP2), septin-7 (SEPT7), protein disulfide-isomerase A3 (PDIA3), and syntenin-1. It also included αB-crystallin and galectin-1, which have anti-inflammatory effects (Wei et al., 2019). Of note, these data on SC exosome content differ from their CNS counterparts, which may, at least in part, explain the higher remyelination efficiency in the PNS.

Oligodendrocyte myelination is regulated by molecules released by other glial cells such as microglia and astrocytes (reviewed by Domingues et al., 2016) but the mechanisms involving exosomes are still unknown. Nevertheless, oligodendroglial exosomes were found to be efficiently internalized by macropinocytosis by non-activated microglia, both *in vitro* and *in vivo*. Significantly, inflammatory stimuli decreased microglia internalization (Fitzner et al., 2011), suggesting that this immunologically silent internalization may represent a clearance mechanism to regulate myelin maintenance under homeostatic conditions.

**FIGURE 1:**
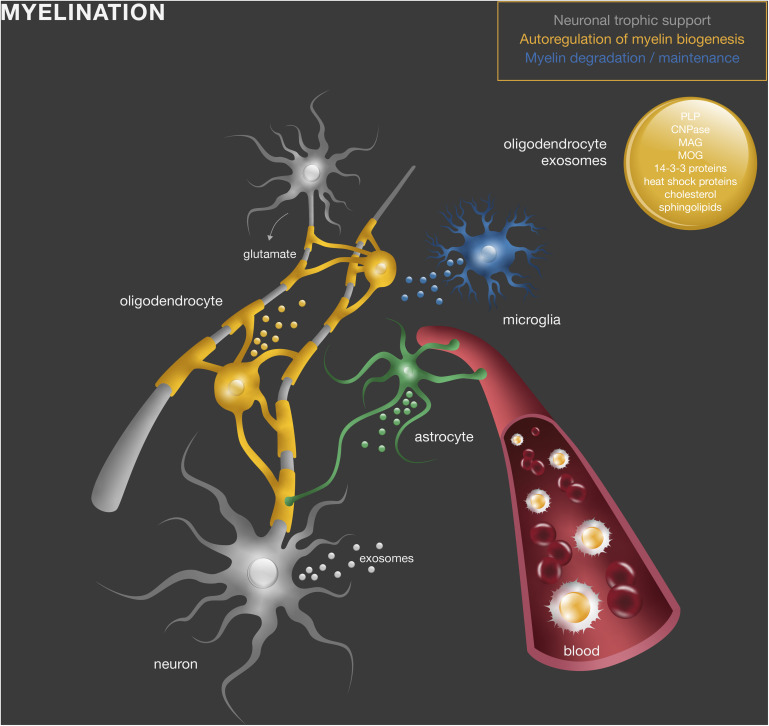
Exosomes in normal myelin development and maintenance. Oligodendrocyte-derived exosomes contain myelin proteins and lipids, including PLP, CNPase, MAG, MOG, cholesterol, and sphingolipids, as well as other molecules such as heat shock and 14-3-3 proteins. They act directly in other oligodendrocytes (yellow), neurons (gray), or microglia (blue) to regulate myelin membrane biogenesis, degradation and maintenance and provide neuronal trophic support.

Altogether, these studies suggest that exosomes produced by myelinating cells convey mechanisms of (1) storage of myelin components for myelin membrane biogenesis, whose release is regulated by neuronal stimuli; (2) transfer of trophic and survival factors to surrounding axons under homeostatic and stress conditions to support effective myelination; and (3) myelin membrane maintenance (Figure 1).

## Exosomes in Demyelination-Associated Conditions: Conceivable Biomarkers of Diagnostics and Disesase Progression

If the content of exosomes is cell-specific and varies accordingly with the biological process in homeostasis, the same logical reasoning applies to the state of disease or injury. Indeed, exosome content changes in response to disease (Manterola et al., 2014; Fiandaca et al., 2015) and is able to cross the blood–brain barrier (BBB) bidirectionally, especially in disease conditions (Zhuang et al., 2011; Chen et al., 2016; Picciolini et al., 2018). Also, their cargo is protected against degradation and can be easily obtained from the patient’s body fluids such as peripheral blood serum and cerebrospinal fluid (CSF) (Street et al., 2012; Kanninen et al., 2016), making them potential candidates for biomarker discovery in diagnostics and disease monitoring.

Demyelination is most commonly associated with the autoimmune disease multiple sclerosis (MS), but it also occurs in several neurodegenerative diseases and conditions such as leukodystrophies, spinal cord injury (SCI), psychiatric disorders, Alzheimer’s, multiple system atrophy (MSA), and cognitive-associated aging (Kremer et al., 2016). Numerous studies have demonstrated a direct association between exosomes and conditions affecting myelin.

Neuroinflammation is one of the most common triggers of myelin loss (Love, 2006). In acute disseminated encephalomyelitis, a brief but intense immune attack to the brain and spinal cord leads to myelin damage. In a similar way, a dysregulated immune-system infiltrates the CNS, causing the destruction of myelin observed in MS and in neuromyelitis optica spectrum disorder (NMOSD), which was originally thought to be a type of MS. The discovery of a specific autoimmunity against a water channel, aquaporin 4, in NMOSD patients that was absent in MS patients made the distinction between these two diseases possible (Lennon et al., 2005). MS has a complex diagnosis as it does not rely on a single diagnostic test but instead on a combination of clinical evaluations, brain imaging techniques such as magnetic resonance imaging (MRI), and complementary tests to exclude other diagnostic possibilities (Gelfand, 2014). To avoid a delay in MS diagnosis, patients would benefit from improved diagnostic biomarkers. This would allow an early and adequate treatment that would impact on the disease course (e.g., by preventing the evolution to a chronic stage). Moreover, the existence of prognostic and treatment-response biomarkers would be crucial for therapeutic decision-making. Despite the progress made over the last years, the discovery of good MS biomarkers is still challenging (Paul et al., 2019).

In the past years, it has been shown that extracellular vesicles can be therapeutic targets and markers of MS. For instance, in rodents with experimental autoimmune encephalomyelitis (EAE), an MS model, vesicles of myeloid origin including exosomes, was increased in the CSF of EAE animals and associated with proinflammatory signals. Impairment of microvesicle release using a transgenic mouse provided resistance to the disease. Interestingly, these microvesicles were also increased in the CSF of patients with neuroinflammation, including MS and clinically isolated syndrome (CIS) patients, when compared to healthy controls (Verderio et al., 2012). Another study showed that the number of CD31 + microparticles of endothelial origin was high in the plasma of MS patients in the exacerbation disease period but not in the remission phase, suggesting that CD31 + microparticles may reflect a chronic injury of the endothelium and represent a marker of acute injury in MS (Minagar et al., 2001). Furthermore, they are thought to bind and activate monocytes and this conjugate is also increased in MS (Jy et al., 2004; Jimenez et al., 2005). Although all these studies refer to extracellular vesicles or microparticles and not specifically to exosomes, these could also comprise exosomes. Likewise, in a study performed in 1989, the authors described the presence of “membrane-attack complex-enriched vesicles” in the CSF of MS patients that are released by viable oligodendrocytes as a protective mechanism (Scolding et al., 1989). This was possibly the first study reporting MS-triggered exosome release. More recently, a correlation was established between exosomes and modulation of MS disease severity. A surprising work demonstrated that during pregnancy, disease progression was halted by exosomes derived from maternal serum, exerting an immunosuppressive effect (Gatson et al., 2011). Moreover, these exosomes induced neuroprotection by promoting migration and maturation of oligodendrocyte precursor cells (OPC) (Williams et al., 2013). This finding could contribute to the observed suppression of MS in pregnant patients.

Alterations in the content of exosomes in response to disease may be due to differences in proteins, lipids, and/or RNA composition. Proteomic analysis of exosomes collected from the CSF of patients with MS, NMOSD, and idiopathic longitudinally extensive transverse myelitis revealed a “biosignature” specific for the three diseases (Lee et al., 2016). Serum exosomes were also shown to carry myelin proteins, such as MBP, PLP, and MOG, both in healthy controls and in MS patients. The levels of MOG were increased both in serum and CSF of MS patients when compared to controls and correlated with the disease severity (Galazka et al., 2018). This suggests that exosomes might contribute to the autoimmune reaction against myelin proteins in MS and may provide novel markers of disease activity.

Other MS-specific exosomal-derived biomarkers and mechanisms of disease have been unveiled by using a lipidomics approach. Pieragostino et al. (2018) demonstrated that exosome-derived lipids are also changed in response to MS and proposed to use CSF acid sphingomyelinase (SMase) as a biomarker of MS as its enzymatic activity was correlated with the disease progression.

Additional exosomal biomarkers in MS are emerging from transcriptomic analysis studies. Serum exosomes carry unique microRNA profiles in relapsing remitting MS, revealing potential diagnostic biomarkers to distinguish MS relapse (Selmaj et al., 2017). Furthermore, distinct circulating microRNAs were identified in relapse and remitting and secondary or primary progressive MS, suggesting that exosomal microRNAs can also predict MS subtypes (Ebrahimkhani et al., 2017). Furthermore, microRNAs from exosomes could be used to monitor therapy response in MS patients. Manna et al. (2018) isolated circulating exosome-derived microRNAs from patients before and after receiving IFN-beta treatment and found 16 microRNAs differentially expressed, suggesting that these microRNA signatures could be used as prognostic biomarkers and to monitor patient responses to therapy. Finally, a recent study described that regulatory T cells were suppressed by circulating exosomes in MS, and this effect was mediated by let-7i, a microRNA that is enriched in exosomes from MS patients (Kimura et al., 2018).

In other CNS diseases with less extensive demyelination, there is also some evidence for the involvement of exosomes but much still remains unexplored. In MSA, an α-synucleopathic neurodegenerative disease, misfolded α-synuclein is accumulated in glial cytoplasmic inclusions (GCIs) of oligodendrocytes. McCormack et al. (2019) suggested that exosomes mediate the spreading of pathogenic unfolded α-synuclein. These were released extracellularly from the presynaptic terminal, taken up by oligodendrocytes and selectively targeted to an inclusion body via retrograde vesicular transport (McCormack et al., 2019).

**FIGURE 2:**
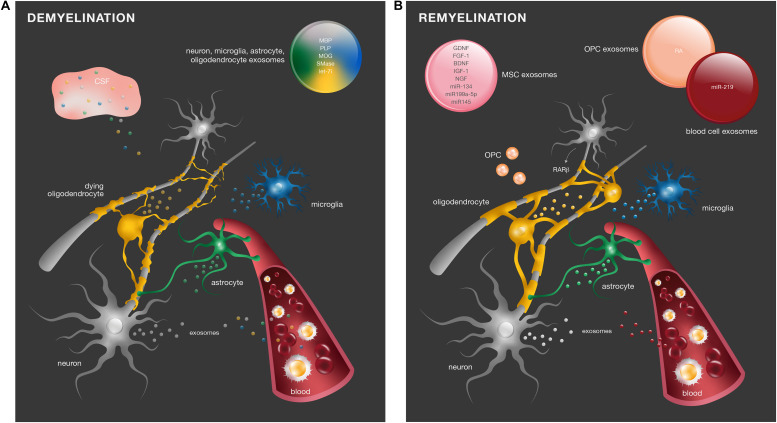
Exosomes in demyelinating diseases and myelin repair. **(A)** In response to myelin damage, CNS resident cells change the content of their exosomes. Differential levels of molecules such as MBP, PLP, MOG, SMase, and the miRNA let-7i have been identified in peripheral blood serum and CSF of demyelinating diseases and may be considered potential diagnostic biomarkers. **(B)** In SCI, retinoid signaling is an important endogenous mechanism for remyelination. RA was identified in exosomes from OPC (orange) to promote neurite outgrowth. Numerous regenerative molecules were found in the exosomes of MSCs (pink), including GDNF, FGF-1, BDNF, IGF-1, NGF, miR-134, miR-199a-5p, and miR-145, with promising therapeutic effects in remyelination.

Altogether, these studies show that there are in fact alterations in exosome content in different demyelinating diseases, and these may be potential disease biomarkers (Figure 2A). However, due to the fact that exosomes are isolated from blood and CSF, it is not possible to assure the exact cell origin from which these exosomes derive.

## Remyelinating Therapies Based on Exosome-Mediated Mechanisms

In SCI, axonal regeneration precedes remyelination, and retinoid signaling is important in this process. It was recently shown that activation of neuronal retinoic acid receptor-beta (RARβ) in neurons promotes synthesis of OPC retinoic acid (RA), which is released in exosomes to act as a positive cue to axonal and dendrite outgrowth (Goncalves et al., 2018). Additionally, RA signaling promotes remyelination after SCI through the synergic interaction of neuron–glia network pathways via exosomes. RARβ agonist treatment of SCI animals led to an increased synthesis and secretion of decorin by neurons. Decorin had a promyelinating effect in NG2 positive OPC by reducing intracellular calcium concentrations, which restricted exosome release and generated an intracellular pool of retinoic acid, promoting OPC differentiation into myelinating oligodendrocytes (Goncalves et al., 2019). These data suggest that remyelination mechanisms via exosomes are a recapitulation of developmental myelination, as discussed above.

Exosomes derived from the blood serum can also have a positive impact on CNS remyelination. Pusic and Kraig showed that blood circulating cells from young and environmentally enriched animals – characterized by increased physical, intellectual, and social activity – secrete exosomes with myelinating and remyelinating effects. They showed that exposure of hippocampal organotypic slice cultures from aged animals to such exosomes promoted OPC differentiation into myelin producing cells, both under normal conditions and after acute lysolecithin-induced demyelination. Additionally, intranasal administration of exosomes to aging naïve rats also enhanced myelination. These effects were mediated by miR-219, highly enriched in peripheral exosomes (Pusic and Kraig, 2014). The authors found that various peripheral cell types produced miR-219-containing exosomes (Pusic et al., 2016). In particular, the same pro-myelinating and remyelinating effects were obtained with exosome treatment from dendritic cell cultures stimulated with low levels of the cytokine IFNγ. These exosomes had a direct impact on myelination as they were preferentially taken up by oligodendrocytes (Pusic et al., 2014). These data provide supporting evidence that cells from youthful and enriched environments release exosomes with regenerative effects in myelination (Figure 2B).

## Therapeutic Potential of MSCs Derived Exosomes and Secretome in Neuroregeneration and Myelination

Mesenchymal Stem Cells (MSCs) secretome is, nowadays, at the forefront of a new wave of possible therapeutic strategies for CNS repair and regeneration (Teixeira and Salgado, 2020).

We have demonstrated that treatment with the secretome of MSCs from bone marrow (BM-MSCs) has promising effects in a rat pre-clinical model of Parkinson’s disease, where sole administration leads to significant motor improvements (Teixeira et al., 2017; Mendes-Pinheiro et al., 2019; Vilaça-Faria et al., 2019; Teixeira et al., 2020). *In vitro*, we have demonstrated positive effects of MSCs secretome in neuronal and glial survival and differentiation and axon growth (Salgado et al., 2010; Teixeira et al., 2017; Assunção-Silva et al., 2018; Mendes-Pinheiro et al., 2019). In the past years, we have been characterizing MSCs secretomes of different sources (Pires et al., 2016) as complex mixtures of soluble components that comprise a proteic soluble fraction (containing trophic and neurotrophic factors), and a vesicular portion composed by microvesicles and exosomes, which can be involved in the transfer of proteins, peptides, and genetic material (e.g., miRNAs) to neighbor cells, and that have revealed important functions in neurodevelopment such as neurogenesis, neurodifferentiation, axonal guidance, and growth (Teixeira et al., 2016, 2017; Martins et al., 2017; Assunção-Silva et al., 2018). Actually, from peripheral to central, and from traumatic to neurodegenerative conditions, MSCs paracrine activity has shown regenerative effects with promising insights to future clinical applications in traumatic brain injury, Parkinson’s disease, MS, or SCI (Ferreira et al., 2018; Praveen Kumar et al., 2019). Notwithstanding, under the context of developmental myelination or demyelination-associated diseases, the concept of MSCs secretome applications still remains to be addressed. As discussed above, exosome-mediated signaling from various cell sources contributes not only to support neuronal functions but also to modulate OPC and their myelin production process (Holm et al., 2018). Therefore, it is plausible that the MSCs secretome has additional positive effects in oligodendrocyte development. Few recent studies have already shed some light on the potential effects that MSC-derived vesicles may have in myelination and oligodendrocytes activity (Laso-García et al., 2018; Dong et al., 2019; Osorio-Querejeta et al., 2019; Thomi et al., 2019). For instance, in perinatal brain injury (PBI), which is characterized by white matter injury and myelination deficits, Thomi et al. (2019) found that intranasal injection of MSC-derived exosomes led to a reduction in neuronal cell death levels and an increase in mature oligodendrocyte counts, thereby rescuing normal gray and white matter neurodevelopment after PBI condition. Based on preliminary findings from deep RNA sequencing data of MSC-derived exosomes, the authors in this study speculated about the potential involvement of certain enriched miRNAs in oligodendrocyte maturation, such as miR199a-5p and miR145. In an *in vivo* subcortical ischemic stroke model, MCS-derived exosomes were shown to promote oligodendrocyte differentiation and remyelination. After intravenous administration, the authors detected higher levels of myelin protein and more myelinated axons (Otero-Ortega et al., 2017). These data are in agreement with an *in vitro* model of ischemic stroke showing that miR-134 derived from exosomes of bone marrow MSCs had a beneficial effect on rat oligodendrocytes by suppressing apoptosis through targeting of caspase-8 (Xiao et al., 2019). In addition to miRNAs, MSC-derived exosomes can also shuttle neurotrophic factors to promote axonal regeneration and tackle peripheral myelin biogenesis, as it is the case of glial cell-derived neurotrophic factor (GDNF), fibroblast factor-1 (FGF-1), brain-derived neurotrophic factor (BDNF), insulin-like growth factor-1 (IGF-1), and nerve growth factor (NGF) (Bucan et al., 2019). Finally, in a model of progressive MS, Laso-García et al. (2018) demonstrated that administration of MSC-derived exosomes from human adipose tissue was able to attenuate motor deficits through immunomodulatory actions, diminishing brain atrophy and promoting remyelination.

Overall, the potential neuroprotective and immunomodulatory actions of MSC-derived vesicles have been explored in the recent past and hold great promise regarding their role in myelination in health and disease (Figure 2B). The content of such nanovesicles suggests multifaceted functions by both regulating neuronal and glial cells behavior. Additionally, their ease of availability places MSCs as favorable sources of vesicles for cell-free therapy to a variety of CNS degenerative diseases, including those affecting myelin. Nevertheless, further characterization and mechanistic studies are still required to address the role of MSC-derived vesicles in developmental myelination and subsequent potential therapeutic applications in the context of demyelinating diseases. More specifically, it is important to identify factors in these vesicles able to modulate OPC migration and axon-oligodendrocyte communication. Ultimately, such knowledge will enable specific reengineering of such nanovesicles with specific loadings for presentation as a biopharmaceutical product to act on demyelinating diseases.

## Conclusion, Challenges, and Opportunities

Myelination is orchestrated by an impressive cooperation between neurons and myelinating glia, which communicate bidirectionally by multiple signals to assure a balance between the necessary number of myelinating oligodendrocytes and the axonal area to be myelinated. All this has to happen in a synchronized manner in order to enable myelination at the proper time and place. Research data described in this review highlight an increasing body of evidence for the relevant role of exosomes in myelin dynamics in health and disease. The surrounding CNS resident glial cells as well as cells from the periphery clearly influence myelination. Still, there is much to be learned about the identity, progeny, and mechanisms of the involved molecules. Other key questions await to be addressed. For example, could exosomes constitute a mechanism of white matter plasticity during adulthood? Do these nanostructures play a role in activity-dependent regulation of myelination during learning? Are exosomes *per se* a potential therapeutic approach or do they require the synergistic collaboration of soluble factors for an effective regenerative effect? One major and ongoing challenge in exosome research is to improve methods of isolation and purification. This would highly benefit the characterization of exosome content in the various contexts, *in vivo* and *in vitro*, in normal and pathological conditions, in animals and humans. In addition, it is highly relevant to understand in more detail exosome biogenesis and secretion mechanisms in CNS cells in order to be able to identify the origin of the different populations and disentangle their physiological relevance for each specific biological process. This fundamental knowledge will be important to decipher the contexts of exosome biology in developmental myelination and, eventually, envision exosomes as potential diagnostic biomarkers and/or therapeutic agents.

## Author Contributions

All authors listed have made a substantial, direct and intellectual contribution to the work, and approved it for publication.

## Conflict of Interest

The authors declare that the research was conducted in the absence of any commercial or financial relationships that could be construed as a potential conflict of interest.
